# Microvascular Muscle vs. Fascio-Cutaneous Free Flaps for Reconstruction of Plantar Load-Bearing Foot Defects—An International Survey

**DOI:** 10.3390/jcm13051287

**Published:** 2024-02-24

**Authors:** Sinan Mert, Paul I. Heidekrueger, Benedikt Fuchs, Tim Nuernberger, Elisabeth M. Haas-Lützenberger, Riccardo E. Giunta, Denis Ehrl, Wolfram Demmer

**Affiliations:** 1 Division of Hand, Plastic and Aesthetic Surgery, LMU University Hospital, LMU Munich, 80336 Munich, Germanywolfram.demmer@med.uni-muenchen.de (W.D.); 2 Centre of Plastic, Aesthetic, Hand and Reconstructive Surgery, University of Regensburg, 93053 Regensburg, Germany; 3 Department of Plastic, Reconstructive and Hand Surgery, Burn Centre for Severe Burn Injuries, Nuremberg Clinics, University Hospital Paracelsus Medical University, 90419 Nuremberg, Germany

**Keywords:** plastic surgery, microsurgery, reconstructive surgery, foot defects, plantar defects, microvascular free flaps, muscle flaps, fascio-cutaneous flaps, survey

## Abstract

**Background**: The reconstruction of plantar load-bearing foot defects faces many plastic surgeons with a major challenge. The optimal patient- and defect-oriented reconstructive strategy must be selected. **Methods**: To analyze the current trends and recommendations in reconstruction of plantar load-bearing foot defects, we conducted an international survey among plastic surgeons querying them about their recommendations and experiences. **Results**: The survey revealed that the most common strategies for reconstruction of the foot sole are locoregional and microvascular free flaps, emphasizing the relevance of plastic surgery. Among microvascular free flaps, muscle and fascio-cutaneous free flaps are by far the most frequently used. The target qualities of the reconstructed tissue to be considered are manifold, with adherence being the most frequently mentioned. We observed a noteworthy correlation between the utilization of muscle flaps and a preference for adherence. In addition, we identified a substantial correlation between the usage of fascio-cutaneous free flaps and further target qualities, such as good skin quality and sensitivity. **Conclusions**: Our findings provide insights into the clinical reality and highlight important aspects that must be considered in reconstruction of the weight-bearing areas of the foot providing support in the selection of the appropriate therapy.

## 1. Introduction

The foot, with its complex static structure, enables humans to walk upright and has a profound influence on the overall musculoskeletal system. The integumentary features of the plantar region, especially the skin and weight-bearing components of the sole, are integral in achieving the required elasticity and mechanical robustness to support the body’s weight. Injuries or wounds of the plantar surface can significantly impede daily activities and potentially lead to patient disability [1,2,3].

The glabrous plantar skin displays a highly developed squamous epithelium, with the stratum corneum being thicker than in any other anatomical region of the body. Subcutaneous fat pads act as crucial buffers, efficiently distributing the body’s weight. These adipose tissues are connected to the plantar fascia through robust septa of connective tissue, providing optimal weight dispersion to mitigate plantar pressure and shear forces [4,5]. The sensitivity of the plantar skin acts as a protective mechanism, guarding against potential local injuries or trauma, while its proprioceptive capabilities are essential for maintaining balance control [3,6,7].

Consequently, reconstruction of plantar load-bearing foot defects is a major challenge for many plastic surgeons. The load-bearing areas comprise the heel, the lateral border, and the ball of the foot. Various strategies for reconstruction of the plantar load-bearing surface of the foot have been described, each with its own advantages and disadvantages. These include conservative treatment, skin grafts, locoregional flaps, and microvascular free tissue transfer. However, in cases of extensive defects, reconstruction using microvascular free flaps is often the only viable option. In these cases, microvascular muscle and fascio-cutaneous free flaps are frequently used [8]. Currently, there is no established gold standard for reconstruction of plantar load-bearing foot defects using microvascular free flaps [8,9,10,11].

This study conducted an international online survey, querying plastic reconstructive surgeons from different countries about their opinions on reconstruction of plantar load-bearing foot defects. The findings provide insights into the clinical reality and highlight attributes considered particularly important when reconstructing plantar defects of the foot. Consequently, it can aid surgeons in the decision-making process.

## 2. Materials and Methods

To analyze current trends in reconstruction of plantar load-bearing foot defects, an online survey consisting of four questions was designed with the online survey tool LimeSurvey (LimeSurvey GmbH, Germany). The survey was conducted completely anonymously in accordance with the general data protection regulation of the European Union. To maximize international participation, the questionnaire was designed in English.

The following four questions were sent to 1205 plastic surgeons from all over the world:

1. How many microvascular free flaps do you approximately operate per year? (Number);
2. How do you treat plantar load-bearing foot defects? (Multiple answers possible);
  - Split or full thickness skin graft;
  - Local and regional pedicled flaps;
  - Microvascular free flaps;
  - Conservative treatment;
  - Other.
3. Which microvascular free flap do you prefer to reconstruct plantar load-bearing foot defects? (Choose the answer that fits the best);
  - Free microvascular muscle flaps;
  - Free microvascular fascio-cutaneous flaps;
  - Free microvascular composite flaps;
  - Other.
4. What is the most important quality of a microvascular free flap used to reconstruct plantar load-bearing foot defects? (Choose the answer that fits the best).
  - Good skin quality;
  - Adherence (no shearing of transplanted tissue);
  - Sensitivity;
  - Other.

Plastic surgeons were contacted by e-mail correspondence through contact listings in national and international specialty societies, including, but not limited to, the member rosters of the American Society of Plastic Surgeons (ASPS), the Brazilian Society of Plastic Surgery (SBCP), the German Society of Plastic, Reconstructive and Aesthetic Surgery (DGPRAEC), the French Society of Aesthetic and Reconstructive Plastic Surgeons (SoFCPRE), the British Association of Plastic Reconstructive and Aesthetic Surgeons (BAPRAS), the Italian Society of Plastic Reconstructive and Aesthetic Surgery (SICPRE), the Spanish Society of Plastic Reconstructive and Aesthetic Surgery (SECPRE), the Oriental Society of Aesthetic Plastic Surgery (OSAPS), the Turkish Society of Plastic, Reconstructive and Aesthetic Surgeons (TPCD), the Korean Society for Aesthetic Plastic Surgery (KSAPS), the Society of Aesthetic Plastic Surgeons of Thailand (ThPRS), the Japanese Society of Plastic and Reconstructive Surgery (JSPRS), and the Indian Association of Aesthetic Plastic Surgeons (IAAPS). The e-mail addresses were sourced from publicly accessible websites of the national societies. The authors lack information regarding completeness and accuracy of the displayed e-mail addresses. Two rounds of reminders were sent after 4 and 8 weeks to non-responders. The survey was closed in September 2023.

The responses were tabulated and descriptive analysis was performed using Microsoft Excel 2019 (Microsoft Corporation, Redmond, Washington, DC, USA). The only exclusion criterion was that only fully completed questionnaires were included in the final analysis. To analyze the correlation between the preferred microvascular free flap (microvascular muscle or fascio-cutaneous free flap), the preferred flap quality, and the number of annually operated microvascular free flaps, the Pearson’s chi-square test and correlation coefficients according to Cramer’s V were calculated using SPSS Statistics 29 (IBM Corporation, Armonk, New York, NY, USA). A *p*-value < 0.05 was considered statistically significant.

## 3. Results

### 3.1. Descriptive Analysis

During a period of 12 weeks, a total of 124 responses (response rate of 10.29%) were gathered. In total, 29 responses (2.41%) had to be excluded due to incomplete answering of the questionnaire, resulting in 95 responses (7.88%) being included in the final analysis. The average number of performed free flap surgeries among the participants was 30.19 ± 3.61 (mean ± standard error of mean) with a median of 15 (question 1).

**Figure 1:**
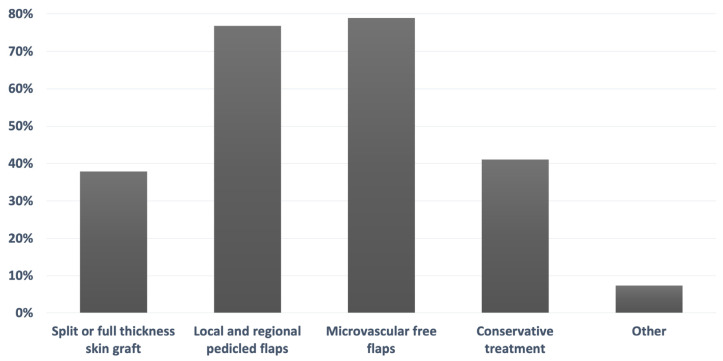
Survey results of the question “How do you treat plantar load-bearing foot defects?” (*n* = 95).

Of the surveyed participants, 37.9% reported treating plantar load-bearing foot defects with split or full thickness skin graft, 76.8% with local and regional pedicled flaps, 78.9% with microvascular free flaps, 41.1% with conservative treatment, and 7.4% with other treatment methods (Figure 1).

**Figure 2:**
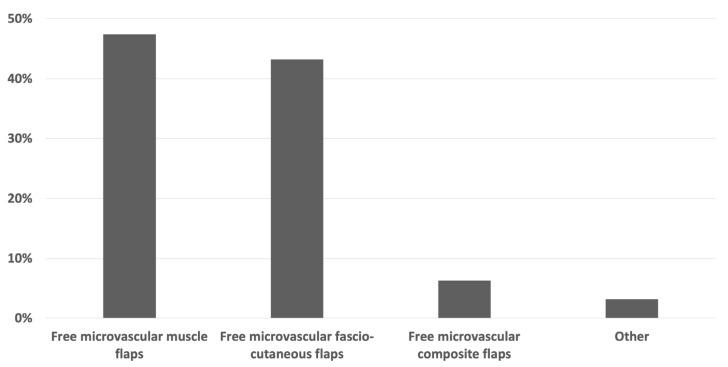
Survey results of the question “Which microvascular free flap do you prefer to reconstruct plantar load-bearing foot defects?” (*n* = 95).

Among the microvascular free flaps for reconstruction of plantar load-bearing foot defects, both free microvascular muscle flaps (47.4%) and free microvascular fascio-cutaneous flaps (43.2%) were the most frequently chosen. Free microvascular composite flaps (6.3%) and other flaps (3.2%) were less commonly selected (Figure 2).

**Figure 3:**
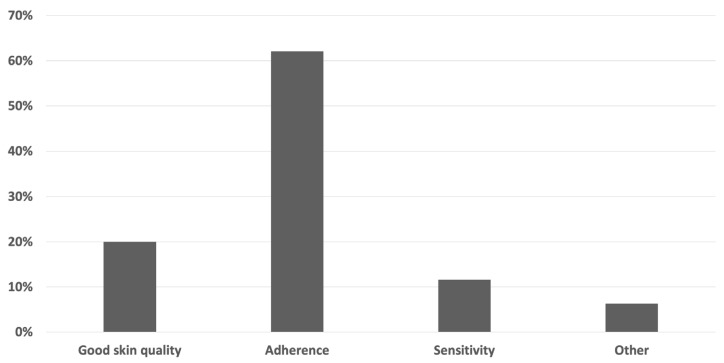
Survey results of the question “What is the most important quality of a microvascular free flap used to reconstruct plantar load-bearing foot defects?” (*n* = 95).

According to the survey results, the most crucial quality of a microvascular free flap in reconstruction of plantar load-bearing foot defects is “Adherence” (62.1%), followed by “Good skin quality” (20.0%), “Sensitivity” (11.6%), and “Other” (6.3%) (Figure 3).

### 3.2. Microvascular Muscle or Fascio-Cutaneous Free Flap?

**Figure 4:**
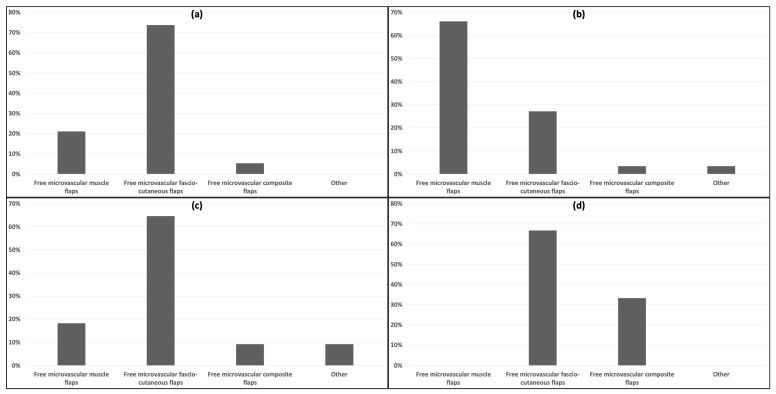
Preferred microvascular free flap among participants who indicated “Good skin quality” (**a**) (*n* = 19), “Adherence” (**b**) (*n* = 59), “Sensitivity” (**c**) (*n* = 11), or “Other” (**d**) (*n* = 6) as the most important quality of a microvascular free flap to reconstruct plantar load-bearing foot defects.

To explore the optimal microvascular free flap for specific flap quality outcomes, a detailed analysis of questions 3 and 4 was conducted. The preferred microvascular free flap was assessed among survey participants who selected “Good skin quality”, “Adherence”, “Sensitivity”, or “Other” (Figure 4). Among the 19 survey participants who considered “Good skin quality” as the most crucial flap quality in reconstruction of plantar load-bearing foot defects, 14 (73.7%) plastic surgeons preferred fascio-cutaneous free flaps, 4 (21.1%) preferred muscle free flaps, and only 1 (5.3%) plastic surgeon favored composite free flaps. Out of the 59 survey participants prioritizing “Adherence” as the most important quality, 39 (66.1%) plastic surgeons favored muscle free flaps, while 16 (27.1%) preferred fascio-cutaneous free flaps. Only 2 (3.4%) plastic surgeons opted for composite free flaps or other. In terms of “Sensitivity”, once again, fascio-cutaneous free flaps were preferred most frequently (7/11 plastic surgeons, 64.6%), while muscle free flaps (2/11 plastic surgeons, 18.2%), composite free flaps (1/11 plastic surgeons, 9.1%), and other (1/11 plastic surgeons, 9.1%) were less commonly selected. Among the 6 survey participants who preferred a different flap quality, 4 (66.7%) mentioned fascio-cutaneous free flaps, and 2 (33.3%) mentioned composite free flaps.

To address the question of which microvascular free flap should be preferred in which situation, the correlation between the microvascular muscle and fascio-cutaneous free flap and the preferred flap qualities was computed (Table 1). Composite free flaps and other flaps were excluded from this analysis as they are selected less frequently (Figure 2). The calculation of Pearson’s chi-square and the correlation coefficients according to Cramer’s V revealed a highly significant correlation between muscle free flaps and “Adherence” (0.496, *p* < 0.001), while fascio-cutaneous free flaps significantly correlated with “Good skin quality” (0.310, *p* = 0.004) and “Other” (0.231, *p* = 0.032). Moreover, a positive but non-significant correlation between fascio-cutaneous free flaps and “Sensitivity” was observed (0.206, *p* = 0.056), likely due to the limited number of participants in the survey (*n* = 95 for the overall survey and *n* = 11 for “Sensitivity”). There was no significant correlation between the number of annually operated microvascular free flaps and the preferred free flap (0.620, *p* = 0.318) or the preferred flap quality (0.616, *p* = 0.267).

## 4. Discussion

Defects of the foot can result from various factors such as trauma, infection, cancer, ulceration, or burn injuries, with trauma being the primary cause [9,12,13,14]. Many different strategies for treating these defects are described in the literature. Particularly, large defects in the load-bearing zone of the plantar foot may necessitate reconstructive surgery to restore appropriate soft tissue coverage. Beyond the defect size and depth, the qualities of plantar skin must be considered to achieve a satisfactory functional and aesthetically pleasing outcome. Only a few donor sites are suitable for meeting these demanding reconstructive criteria and their performances may vary. In this study, we conducted an international online survey, querying plastic reconstructive surgeons from different countries about their opinions on reconstruction of plantar load-bearing foot defects, identifying current trends and relevant criteria.

In 2019, Crowe et al. conducted a systematic review on reconstruction of the plantar surface of the foot, analyzing 280 unique articles encompassing a total of 2684 individual reconstructions. Among the reviewed articles, 10% utilized skin grafts, 53% employed locoregional flaps, 32% employed free tissue transfer, and 5% described multiple reconstructive methods [8]. Other methods for reconstruction/therapy include dermal grafting, dermal substitutes, or amputation, for example [15,16]. Our results confirm that locoregional and microvascular free flaps are the most commonly used methods of reconstruction.

In the case of chronic wounds, comprehensive surgical debridement should precede all reconstructive attempts. In selected instances, such as when the patient’s general condition demands, the wound may be left for conservative healing, possibly supported by negative pressure wound therapy (NPWT) [3,8]. Reconstruction with free (split) skin grafting is feasible only in superficial areas without mechanical strain, while load-bearing or subfascial defects necessitate reconstruction with tissue flaps. Adhering to the general principle of reconstructing defects with tissue of equal quality, local flaps like the medial plantar (instep) flap or the reverse sural flap are preferably used [8,17,18,19]. In cases of an impaired donor site on the injured foot, these flaps can also be employed from the contralateral side. Given their limited size or reach, they are suitable only for defects of moderate size [20,21,22]. Further locoregional flaps are, for example, the intrinsic muscle flaps, the toe island, and fillet flaps [8]. For larger plantar defects and in cases where locoregional flaps cannot be used, free microvascular flaps are the method of choice for reconstruction, with the most commonly used being microvascular muscle and fascio-cutaneous free flaps, each having its advantages and disadvantages due to their tissue composition. In selected cases, other microvascular free flaps such as composite and/or chimeric flaps (e.g., in cases of extensive and composite defects of the sole) are required [8,23,24]. For example, the deep circumflex iliac artery flap with a tricortical iliac crest can be used for reconstruction of plantar defects with additional defects in the calcaneal bone. In addition, the sartorius tendon can be used for reconstruction for the Achilles tendon [25,26,27].

Muscle free flaps, particularly the latissimus dorsi and gracilis, are well established for lower limb defect reconstruction [8,10]. Due to the size of the latissimus dorsi, it can provide total plantar coverage [8]. Other muscle free flaps used for reconstruction of plantar load-bearing foot defects include serratus [28], rectus abdominis [29], tensor fascia latae [30,31], and biceps femoris [32], among others. They offer advantages such as a constant vascular supply, low anatomical variability, overall low donor morbidity, and ease of harvesting [33,34]. In plantar reconstruction, muscular free flaps are valued for their tendency to atrophy over time, adapting to the foot’s contour, and their high adherence to the underlying musculoskeletal structures. However, they require coverage with split-thickness skin and lack sensitivity, making them susceptible to ulceration [11].

Fascio-cutaneous free flaps provide good skin quality, since they contain full-fledged anatomical skin with its subcutaneous tissue. Depending on patient-specific characteristics, they display a variable portion of subcutaneous fat tissue. The most commonly used fascio-cutaneous free flap is the anterior lateral thigh (ALT) flap, as first described by Song et al. in 1984 [10,35]. Its relatively bulky nature can be thinned primarily or in a delayed approach without the risk of necrosis [36]. The radial forearm flap is also commonly employed for reconstruction of the foot sole and rarely needs to be debulked due to its thinness. For improved sensitivity, both flaps can be harvested with inclusion of the lateral femoral cutaneous nerve or lateral antebrachial cutaneous nerve, respectively [37,38,39]. Another suitable microvascular fascio-cutaneous free flap is the medial plantar (instep) flap from the contralateral foot. As already mentioned, this flap offers the advantage of similar skin quality with a thick stratum corneum and dense fibrous septa [22,40].

Our results affirm literature findings indicating that microvascular free muscle and fascio-cutaneous free flaps are the most employed for reconstruction of plantar load-bearing foot defects [8].

Increased mobility of a free flap due to its bulkiness can lead to hyperkeratosis and fissures, especially in its peripheral area, resulting in shear effects that potentially cause feelings of instability and may lead to ulceration. Well-contoured muscle flaps with skin graft coverage exhibit this issue to a lesser degree due to their higher adherence to the underlying tissue. Therefore, some authors describe a lower rate of ulceration in plantar reconstruction when using free muscle flaps [41,42,43].

One of the primary distinctions between muscle and fascio-cutaneous free flaps is the character of their skin mantle. While fascio-cutaneous free flaps have a histologically full-fledged (hairy) skin covering, muscle free flaps require coverage through additional skin grafting. Even though the fundamental principle in reconstructive surgery is to replace like with like, long-term studies have not shown a significant disadvantage of split skin grafting in this context [42,44,45]. It should also be noted that the hairy skin mantle of the ALT flap is not completely identical to the skin of the weight-bearing areas of the foot, as already mentioned.

In 2019, Heidekrueger et al. [9] conducted a study on long-term functional outcomes and quality of life after microsurgical reconstruction of the plantar foot. When comparing a total of 100 plantar foot reconstructions (46 with ALT flap and 54 with gracilis muscle flap), no significant differences were observed in major or minor complications. The ALT group exhibited significantly less pain and scarring, along with significantly better superficial and depth sensitivities, as well as shoe provision. However, there was a significantly higher requirement for secondary surgeries in the ALT group, mostly for flap debulking.

The necessity to reinnervate the transplanted tissue is still a subject of debate [9,44,46,47,48]. The objective is to reestablish sensory perception and, thereby, protect the flap from damage due to unnoticed overuse or pressure points. Several studies have provided evidence on reinnervation of the free flap after coaptation to a donor nerve in the recipient region, with fascio-cutaneous free flaps being predominantly used. As already mentioned, the ALT and radial forearm flap allow nerve coaptation of their sensory nerves [37,38,39]. Nevertheless, sensory recovery has also been reported in muscle flaps after reinnervation of the flaps’ motor nerve by a sensory nerve [49,50]. However, even without sensory reconstruction, some protective sensation is regained within the first 12 months after free tissue transfer without nerve coaptation [51].

In a study on the donor-site morbidity after free ALT or gracilis muscle flap by Fricke et al. [52], no significant differences were found in functional impairment of the lower extremity. However, the gracilis muscle flap proved to be superior to the ALT flap in terms of scar length and numbness of the donor site.

In clinical defect reconstruction, stem cell-based approaches are assuming an increasingly pivotal role owing to the regenerative potential of human stem cells, which encompass proliferative, immunomodulatory, and angiogenetic properties. Multiple studies have underscored the beneficial impact of adipose-derived stem cells (ASCs) on the healing process of diabetic foot ulcers [53]. Notably, Raposio and Bertozzi have described a method facilitating the ready-to-use isolation of ASCs tailored for clinical application [54]. This innovation not only opens avenues for augmenting defect coverage through flee flap surgery but also underscores the potential of ASCs in clinical settings. However, further studies and surveys are imperative to determine the full scope of stem cell application and facilitate its seamless integration into clinical practice.

The aim of our study was to identify the relevant criteria in the reconstruction of plantar load-bearing foot defects and draw conclusions regarding the most suitable reconstruction method. In this context, we focused on the most frequently mentioned target qualities—adherence, good skin quality, and sensitivity. Other relevant criteria include aesthetic patient satisfaction, shoe provision, frequency and severity of pain at the recipient and donor sites, and donor-site morbidity. The results of our study indicate that adherence (no shearing of transplanted tissue) is by far the most important quality of a microvascular free flap to reconstruct plantar load-bearing foot defects. The experience of the surgeons had no significant influence on the preferred reconstruction method. It is important to note that the evidence obtained in this study is based on an international survey among 95 plastic surgeons and the analysis of the correlation of their answers. To gain further evidence regarding causality, further prospective investigations are required.

## 5. Conclusions

Our research highlights the diverse array of strategies and considerations involved in the reconstruction of plantar load-bearing foot defects. Notably, none of the strategies fulfill the requirements for all patients and defects. However, for large defects, reconstruction using microvascular free flaps becomes indispensable, with muscle and fascio-cutaneous free flaps being the most commonly used.

While the factors to be considered are diverse, adherence is by far the most frequently mentioned target quality, showing a significant correlation with the use of muscle free flaps. Concerning other qualities, particularly good skin quality, sensitivity, and aesthetics, there is a positive correlation with fascio-cutaneous free flaps. Additionally, the expertise and experience of the surgeon must also be taken into account when choosing the appropriate technique.

## Figures and Tables

**Table 1:** Statistical analysis of the correlation between preferred microvascular free flap (question 3) and preferred quality of a microvascular free flap (question 4) to reconstruct plantar load-bearing foot defects.

Microvascular Free Flap	Quality	Pearson’s Chi-Square	Correlation Coefficient
Muscle	Good skin quality	8.27 (*p* = 0.004)	−0.310
Fascio-cutaneous			0.310
Muscle	Adherence	21.12 (*p* < 0.001)	0.496
Fascio-cutaneous			−0.496
Muscle	Sensitivity	3.65 (*p* = 0.056)	−0.206
Fascio-cutaneous			0.206
Muscle	Other	4.60 (*p* = 0.032)	−0.231
Fascio-cutaneous			0.231

## Data Availability

The data presented in this study are available on request from the corresponding author.
